# Interventions for improving adherence to psychological treatments for common mental disorders: a systematic review

**DOI:** 10.1017/gmh.2024.94

**Published:** 2024-10-17

**Authors:** Bijayalaxmi Biswal, Yashi Gandhi, Daisy R. Singla, Richard Velleman, Brian Zhou, Luanna Fernandes, Vikram Patel, Matthew Prina, Miriam Sequeira, Ankur Garg, Urvita Bhatia, Abhijit Nadkarni

**Affiliations:** 1 Addictions and Related Research Group, Sangath, India; 2 Department of Population, Addictions and Related Research Group, Sangath, India; 3 Addictions and Related Research Group, Centre for Addiction and Mental Health, Toronto, ON, Canada; 4 Department of Psychiatry, University of Toronto, Toronto, ON, Canada; 5 Department of Anthropology, Harvard University, USA; 6 Department of Global Health and Social Medicine, Harvard Medical School, Boston, Massachusetts, MA; 7 Population Health Sciences Institute, Faculty of Medical Sciences, Newcastle University, Newcastle Upon Tyne, UK; 8 Department of Population Health, Centre for Global Mental Health, London School of Hygiene and Tropical Medicine, London, UK

**Keywords:** adherence, psychotherapy, interventions, depression, anxiety

## Abstract

Our systematic review aims to synthesise the evidence on interventions targeting improvement in patient adherence to psychological treatments for common mental disorders. A search was conducted on six electronic databases using search terms under the following concepts: common mental disorders, adherence, psychological treatments and controlled trial study design. Due to the heterogeneity in intervention content and outcomes evaluated in the included studies, a narrative synthesis was conducted. Risk of bias was assessed using the Cochrane Risk of Bias Version 2 tool for randomised controlled trials and the Cochrane ROBINS-I tool for non-randomised controlled trials. The search yielded 23 distinct studies with a total sample size of 2,779 participants. All studies were conducted in high-income or upper-middle-income countries. Interventions to improve patient adherence to psychological treatments included reminders and between-session engagement (e.g., text messages), motivational interviewing, therapy orientation (e.g., expectation-setting) and overcoming structural barriers (e.g., case management). Interventions from 18 out of 23 studies were successful in improving at least one primary adherence outcome of interest (e.g., session attendance). Some studies also reported an improvement in secondary outcomes – six studies reported an improvement in at least one clinical outcome (e.g., depression), and three studies reported improvements in at least one measure of well-being or disability (e.g., days spent in in-patient treatment). By incorporating these interventions into psychological treatment services, therapists can better engage with and support their patients, potentially leading to improved mental health outcomes and overall well-being.

## Impact Statement

Despite the high prevalence of common mental disorders and the availability of evidence-based interventions, access to treatments remains limited and adherence rates are often suboptimal, posing significant challenges to improving mental health outcomes worldwide.

Our findings underscore the potential of adherence-focused interventions not only to enhance treatment engagement but also to yield positive clinical outcomes, contributing to overall improvements in mental health and well-being. By integrating these strategies into psychological treatment services, we hope that therapists can better engage with and support their patients, potentially leading to improved mental health outcomes on a global scale. Additionally, the review discusses various gaps in evidence like the lack of focus on non-patient-related dimensions of adherence (e.g., social, economic, health systems related), the absence of studies conducted in low- and middle-income countries and the scope of scalability or wider implementation of the effective interventions.

## Background

Common mental disorders (CMDs), including depression, anxiety and somatoform disorders, are among the leading causes of disability worldwide, accounting for 23% of all years lived with disability (Whiteford et al., 2016). The Global Burden of Disease study estimates that depressive and anxiety disorders accounted for the largest proportions of disability-adjusted life-years (DALYs) related to mental disorders in 2019, contributing 37% and 23% of the total DALYs, respectively (Murray, 2022). Evidence-based psychological treatments – spanning cognitive, behavioural and interpersonal treatment elements – are effective in treating these disorders across a wide range of contexts (Patel et al., 2016). While psychological treatments generally have significant therapeutic effects (Singla et al., 2017; Munder et al., 2019), the duration and frequency of contact required for clinically meaningful improvement can vary depending on the treatment (Hansen et al., 2002; Robinson et al., 2020).

According to the World Health Organisation (WHO), patient adherence is defined as “the extent to which a person’s behaviour – taking medication, following a diet and/or executing lifestyle changes – corresponds with agreed recommendations from a healthcare provider” (Sabaté, 2003). The WHO’s Adherence to Long-Term Therapies Project outlines five dimensions that impact patient adherence: (1) social- and economic-related factors (e.g., poverty, illiteracy); (2) health system and healthcare team-related factors (e.g., short consultations, overworked healthcare workers); (3) therapy-related factors (e.g., previous treatment failures, immediacy of beneficial effects); (4) condition-related factors (e.g., severity of symptoms, level of disability) and (5) patient-related factors (e.g., anxiety about possible adverse effects, low motivation) (Sabaté, 2003). These dimensions challenge the traditional notion that patient adherence is solely a patient-driven problem and provide a framework to guide the design and implementation of strategies for promotion of adherence to long-term therapies.

Patient adherence to a minimally effective dose of therapy remains challenging; 20–47% patients drop out from therapy before recovering from the mental health problems that first led them to seek care (Wierzbicki and Pekarik, 1993; Swift and Greenberg, 2012; Fernandez et al., 2015). Apart from reduced treatment efficacy, poor patient adherence to psychological treatment also leads to empty appointment slots and inefficient resource allocation, ultimately resulting in the reduced cost-effectiveness of providing mental health services (Barrett et al., 2008; Bosworth, 2010). Furthermore, receiving a suboptimal ‘dose’ of psychological treatment may exacerbate symptoms, leading to either increased or decreased future use of mental health services than would have otherwise occurred (Walitzer et al., 1999). Finally, poor patient adherence can result in therapist burnout and workforce turnover by increasing workload and wastage of time (Pekarik, 1985; Piper et al., 1999). These inefficiencies tend to have the highest impact in community settings where human and financial resources are already limited (Walitzer et al., 1999; Oldham et al., 2012).

Previous reviews of interventions to improve patient adherence to psychological treatments were not systematic and examined observational or case studies (Walitzer et al., 1999; Barrett et al., 2008; Bosworth, 2010); and those that systematically synthesised RCTs of such interventions are now more than 10 years old (Oldham et al., 2012). Additionally, most reviews examining patient adherence have disproportionately focused on interventions to enhance psychotropic medication adherence for serious mental illness (Barkhof et al., 2012; Steinkamp et al., 2019). In contrast, the primary aim of our review is to systematically examine the existing literature on interventions targeting patient adherence to psychological treatments for CMDs. Specifically, our objectives are to (a) synthesise evidence on the effectiveness of these interventions in enhancing patient adherence to psychological treatment; (b) describe the implementation (content, delivery methods, acceptability, feasibility) of these interventions and (c) summarise the secondary impact of these interventions on clinical outcomes related to mental health symptoms, well-being, disability and quality of life.

## Methods

Our systematic review is reported in accordance with the Preferred Reporting Items for Systematic Reviews and Meta-Analyses statement (Page et al., 2021). The protocol was developed a priori and registered on PROSPERO (CRD42021266680).

### Eligibility criteria

Peer-reviewed primary research publications in English were included. There were no restrictions on geographical location or year of publication. Eligible study populations included adults (>18 years) diagnosed with any type of CMD (depression, anxiety and somatoform disorders) (Lund et al., 2010). Only randomised controlled trials (RCTs) and non-RCTs (nRCTs) were included.

An intervention was eligible if it was designed and tested specifically for improving patient adherence to a psychological treatment for CMDs. Interventions designed solely to enhance treatment initiation were excluded as patient non-adherence by failing to continue or complete psychological treatment is a distinct construct from that of rejecting psychological treatment, seen when an individual does not attend their initial treatment session (Swift and Greenberg, 2012). Interventions not designed or evaluated to directly target patient adherence to a psychological treatment, but which led to improved adherence as an indirect/secondary outcome or psychological treatments with adherence components within them, were excluded. Interventions designed or evaluated to target adherence to pharmacological treatment or digital psychological treatment, were excluded as they are fundamentally different in nature (Ludden et al., 2015; Gast and Mathes, 2019; Lippke et al., 2021).

The primary outcome of interest was adherence to any kind of psychological treatment, that is, individual or group, delivered independently or in combination with pharmacological treatments, and delivered in-person or by humans via telecommunication technologies (e.g., tele-counselling). Given our definition of patient adherence, studies that measured adherence outcomes after the completion of an initial session of psychological treatment up until the completion of a final session, defined by either the fixed endpoint of a treatment protocol or by mutually agreed discharge between patient and provider, were considered. The primary outcome of interest was any objective measure of patient adherence to psychological treatment, including appointment attendance, homework compliance and indicators of treatment adherence (such as uptake, engagement, motivation, utilisation, participation, completion or retention) or nonadherence (such as treatment discontinuation, dropout, withdrawal, attrition, interruption or premature termination). Secondary outcomes of interest were clinical outcomes related to mental health symptoms, well-being, disability, and quality of life and implementation outcomes.

### Search strategy

Six electronic databases were searched: MEDLINE, PsycINFO, Embase, Global Health, the Cumulative Index to Nursing and Allied Health Literature and the Cochrane Central Register of Controlled Trials. The search was first conducted in July 2021 and subsequently updated in February 2023, using search terms under the following concepts: CMDs (e.g., “major depressive disorder”), patient adherence (e.g., “compliance”), psychological treatments (e.g., “cognitive behavioural therapy” [CBT]) and controlled trial study design (e.g., “RCT”). The detailed search strategy for MEDLINE can be found in Supplementary Material, Appendix 1.

### Study selection and data extraction

Search results from all electronic databases were merged and imported into EndNote for removal of duplicates. After automatic and manual de-duplication, the remaining papers were imported to Covidence, an online software for managing systematic reviews. Papers were also manually screened for duplicates on the Covidence platform. Two pairs of reviewers (SR and RB, BB and LF) independently screened all titles and abstracts as well as full texts for eligibility; and conflicts were resolved by a third reviewer (AG or AN). For the title and abstract screening, consensus was reached for 93% and 95% of publications for the two pairs of reviewers, respectively. For the full-text screening, consensus was reached for 89% and 91% of studies for the two pairs of reviewers respectively.

Forward and backward citation chaining of included studies was conducted using the Web of Science and Google Scholar to identify any additional eligible studies not identified by our primary search. A data extraction form was developed *a priori* on MS Excel to collect data relevant to the objectives of this review. Data were extracted by two pairs of researchers (SR and RB, BB and LF). Inter-rater reliability among raters for data extraction, as measured by Cohen’s Kappa was κ=0.81 and κ=0.84 for the two pairs, respectively.

### Data analysis

Due to the heterogeneity in intervention content and outcomes evaluated in the included studies, a narrative synthesis was conducted (Popay et al., 2006). This involved a descriptive analysis of the studies included in the systematic review, using a textual approach to summarise and explain the results of the synthesis (Popay et al., 2006). Intervention components were categorised under common themes and the content and effectiveness outcomes were also described. These are presented in a tabular format in Supplementary Material, Appendix 2. Delivery methods, clinical outcomes and implementation outcomes were summarised under separate categories (Proctor et al., 2013).

### Quality assessment

Risk of bias was assessed independently using the Cochrane Risk of Bias Version 2 (RoB 2) tool for RCTs and the Cochrane ROBINS-I tool for nRCTs (Sterne et al., 2016; Sterne et al., 2019). The domains included in RoB 2 are bias arising from the randomisation process, deviations from intended interventions, missing outcome data, measurements of the outcome and selection of the reported result. The domains included in ROBINS-I are bias due to confounding, selection of participants, classification of interventions, deviations from intended interventions, missing data, measurement of the outcome and selection of the reported result. To mitigate subjectivity in assessment, two reviewers (BZ, BB) independently answered signalling questions embedded in the ROBINS-I Microsoft Word template and the RoB 2 Excel tool, which uses an algorithm to generate an overall risk of bias based on ratings in each domain. Both reviewers arrived at highly similar ratings in the rating of each domain and overall risk of bias; disagreements were discussed until consensus was reached.

## Results

**Figure 1.**
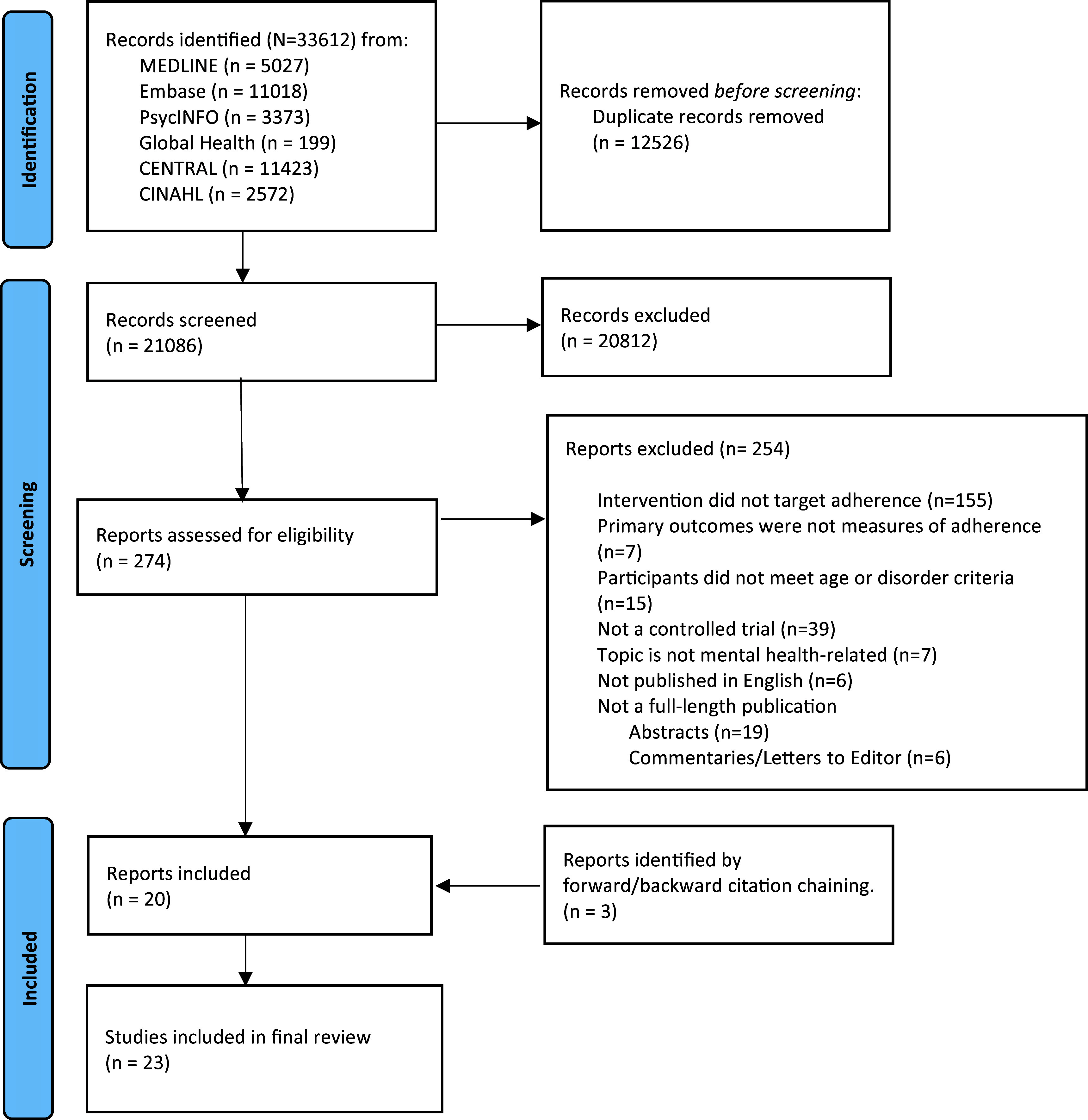
Identification of studies via databases and registers.

Figure 1 summarises the results of our search. Of the 33,612 reports identified, 12,526 were duplicates. From the remaining, 20,812 papers were excluded at the title and abstract screening stage. In total, 274 full texts were assessed for eligibility and based on our criteria, 20 studies were eligible for inclusion. The forward and backward citation chaining process identified three additional eligible studies, leading to 23 studies being included in our review.

### Study characteristics

The 23 included studies comprised a total sample size of 2,779 participants, and the sample sizes in the individual studies ranged from 7 to 325 participants. All studies were from high-income or upper-middle-income countries: United States (*n* = 9), United Kingdom (*n* = 4), Australia (*n* = 3) and Canada (*n* = 3), Austria (*n* = 1), Sweden (*n* = 1), China (*n* = 1) and Chile (*n* = 1). Of the total, 18 were RCTs and 5 were nRCTs. 16 of the RCTs were individually randomised trials and two were cluster RCTs (Raue et al., 2019; Wang et al., 2022). The five nRCTs utilised a variety of participant allocation methods, including self-selection (Furber et al., 2014; Wells et al., 2020), propensity score matching (Delgadillo and Groom, 2017), scheduling convenience (Aguilera et al., 2017) and timing of trial enrolment (Daley et al., 1998). Comparators or controls in the studies were usual care, enhanced usual care, another adherence intervention, delayed intervention or wait-list control. The characteristics of these studies are summarised in Table 1.

**Table 1.** Summary characteristics of included studies

First author, year of publication	Country	Study design	*N*	Target group	Psychological treatment	Adherence intervention in addition to psychological treatment	Control	Adherence outcomes	Symptom/functional outcomes
Aguilera et al., 2017	USA	nRCT	85	Low–income, Spanish speaking Latino patients with depression referred to a behavioural health clinician	CBT	Automated text messages including mood rating prompts, reinforcement of therapy content, medication reminders and therapy reminders.	Only CBT	Significant increase in number of weeks in CBT before dropping out 13.5 vs. 3, *p* = .03	No difference in improvement of depression symptoms between both arms
Alfonsson et al., 2019	Sweden	RCT	7	University students with symptoms of moderate stress or anxiety	Relaxation programme	Text messaging adjunct administered by a computer programme that personalises messages and automatically sends prompts to mobile phones.	Only relaxation programme	There was a significant effect of daily text messages on homework adherence across participants with weak to medium effect size improvements. *p* = .018	Not reported
Avishai et al., 2018	United Kingdom	RCT	8	Economically disadvantaged patients referred to a community facility for anxiety and/or depression	Stress control (SC) psychoeducation course	Pre–course mailing containing questionnaire on participants’ views on attendance and an implementation intention (if–then plan) linking an obstacle to attendance with a pre–planned response. Patients were instructed to read and repeat intention 3 times.	Pre–course mailing without an implementation intention	Significant increase in: – Number of psychoeducation sessions attended 2.84 (2.14) vs. 1.62 (1.76), *p* < .01 – Percent of patients attending at least three or five psychoeducation sessions (out of 5) Third session: 59.5% vs. 33.3%, *p* < .06 Fourth session: 43.2% vs. 24.4%, *p* < .12 Fifth session: 62.2% vs. 22.2%, *p* < .06	Not reported
Barrera et al., 2016	USA	RCT	39	Persons with anxiety disorder presenting for treatment at a university anxiety disorder clinic	Transdiagnostic CBT	Motivational interviewing pre–treatment focused on ambivalence and motivation to anxiety change.	Only CBT	Significant increase in: – Number of CBT sessions attended after treatment initiation: 8.85 (2.93) vs. 6.00 (4.93), *p* = .034	No difference in improvement of anxiety symptoms
Clough *et al.*, 2014	Australia	RCT	140	Patients seeking outpatient psychotherapeutic treatment at psychology clinic treating primarily mood and anxiety disorders	CBT	SMS reminders were sent the day prior to scheduled appointments. Messages were in a standard format and included the client’s name, appointment day and time and contact details for the clinic	Only CBT	Significant differences in: –Therapist determination of premature termination during study period: 38 vs. 24, *p* = .032	Not reported
Daley et al., 1998	USA	nRCT	23	Patients with both a depressive disorder and a cocaine dependence disorder and discharged from a university psychiatric hospital	Outpatient psychological treatment for dual diagnoses	Integrated dual disorder recovery counselling and motivational therapy aimed at helping patients anticipate and cope with contributors to poor adherence	Treatment as usual	Significant difference in: – Treatment sessions attended 7.2 (2.4) vs. 2.7 (2.1), *p* < .001 – 30–day and 90–day treatment completion rates (not defined) 11 (100%) vs. 5 (41.7%), *p* = .01 8 (72.7%) vs. 1 (8.3%), *p* = .006	Significant reduction in: – Psychiatric rehospitalisations at 1 year 0.2 (0.4) vs. 1.1 (0.99), *p* = .03 – Number of days spent in inpatient treatment at 1 year 1.9 (4.2) vs. 12.1 (13.3), *p* = .03
Delgadillo et al., 2015	United Kingdom	RCT, 3 arms	254	Patients referred to a community facility for anxiety and/or depression	CBT–based guided self–help intervention	Arm 1 received an initial appointment confirmation packet with a leaflet setting expectations, normalising concerns and creating implementation intentions around attending therapy. Arm 1 also received a text message reminder 2 days before first appointment. Arm 2 received the same appointment confirmation packet without a text message	Appointment confirmation packet without an orientation leaflet or text	No effect on treatment initiation (attending at least one CBT session) and treatment completion (not defined)	Not reported
Delgadillo and Groom, 2017	United Kingdom	nRCT	98	Patients on waitlist for CBT for anxiety and/or depression	CBT	Pre–treatment transdiagnostic seminars delivered in lecture–style format	Only CBT	Significant increase in –Mean number of weeks in CBT 21.00 (14.06) vs. 14.37 (9.24), *p* = .02 –Number of participants who completed therapy (as opposed to dropped out) (87.8%) vs. (68.8%), *p* = .02	Significant difference in improvement of –Anxiety symptoms –0.53 (.63) vs. 1.25 (.63) (SE), *p* = .05
Furber et al., 2014	Australia	nRCT	202	Individuals who present to the emergency department in situational and/or emotional crises but who are not subsequently admitted to psychiatric services	Telephone based psychotherapy	Individually tailored between–session text messaging	Only telephone–based psychotherapy	No effect on treatment length in days, number who completed treatment, mean number of telephone and face–to–face sessions attended and number of recorded “did not attend” sessions	No significant differences in improvement of anxiety symptoms, functional impairment and depression symptoms
Hoehn–Saric et al., 1964	USA	RCT	40	Persons with a psychoneurotic disorder presenting for treatment at a university psychiatric outpatient clinic	Brief psychotherapy	Role induction interview consisting of four components: (i) explanation of psychotherapy, (ii) expected role of patient and therapist, (iii) preparation for typical challenges during therapy and (iv) expectation for time to improvement.	Only psychotherapy	Significant difference in number of sessions attended: 13.8 vs. 11.5, *p* < .02	Significantly better (as reported): – Therapist ratings of global improvement 6.0 vs. 5, *p* < .05 – Patient ratings of target symptom improvement 4.1 vs. 3.4, *p* < .05
Jurinec and Schienle, 2020	Austria	RCT, 3 arms	126	Patients with major depressive disorder referred to a community health centre	*Coping with Depression* CBT–based outpatient course	Arm 1 received a vial of sunflower oil placebo and was told it was a traditional medicine for depression. Instructed to take three drops prior to daily home relaxation exercises for 21 days Arm 2 received group CBT alone. Instructed to practice daily home relaxation exercises for 21 days	Waitlist	Significant increase in number of home relaxation exercises completed when using sunflower oil Placebo group vs. standard group: 17.8 (3.0) vs. 10.6 (5.5), *p* < .001	Significant improvement in improvement of: – depression symptoms placebo group vs. standard group vs. waitlist: 13.40 (8.03) vs. 16.93 (7.92) vs. 22.19 (6.52), *p* < .03
Latour and Cappeliez, 1994	Canada	RCT	26	Older adults (65+) with major depressive disorder	Cognitive therapy	Group sessions aimed at explaining therapy structure, addressing misconceptions about therapy and observing/practicing desired therapy behaviours	Pretherapy group sessions theoretically unrelated to therapy or depression	No effect on dropout rate (not defined), and number of sessions attended	Not reported
Miranda et al., 2003	USA	RCT	199	Publicly insured and indigent patients with major depression referred to a depression clinic	CBT	Case management in patient–identified areas such as housing, employment, social service assistance and interpersonal conflict	Only CBT	Significant difference in dropout rate (completing less than 8 of 12 sessions) 17% vs. 40%, *p* = .03 Significant difference in number of sessions attended. 10.5 vs. 8.4, *p* = .002	Significant difference in improvements of depression symptoms across arm 13.9 vs. 14.2, *p* = .03 Significant difference in improvement in social functioning 53 vs. 50.6, *p* = .03
Mohr et al., 2012	USA	RCT	325	Primary care patients with major depressive disorder	CBT	Telephone–administered CBT	Face–to–face CBT	Significant difference in number of CBT sessions attended 15.5 (4.4) vs. 13.7 (6.1), *p* = .003 Significant difference in: – overall dropout rate (nonresponsive after missing 1 session plus 2 phone calls and a letter) 20.9% vs. 32.7%, *p* = .02 – dropout rate in first four CBT sessions 4.3% vs. 13%, *p* = .006	Face–to–face CBT had significantly greater improvement in depression symptoms at 6 months than telephone CBT difference 2.91 (1.20 to 4.63 CI), *p* < .001
Pérez et al., 2021	Chile	RCT	167	Patients seeking treatment for depression at a private, university–affiliated clinical and research mental healthcare facility	Psychotherapy	ASCENSO is an online programme for monitoring and supporting patients with depression, delivered as adjunctive support to conventional face–to–face treatment	Only CBT	No significant difference in percentage of treatment sessions attended, including appointments for medical control, psychiatry and psychotherapy; and percentage of participants who dropped out from TAU (face–to–face interventions)	No significant difference for improvement in depressive symptoms
Peters et al., 2019	Australia	RCT	186	Persons with social anxiety disorder presenting for treatment at a university mental health centre	CBT	Motivational interviewing pre–treatment focused on ambivalence, motivation, obstacles to changing behaviour and expectations around therapy process and outcome	Pretherapy supportive counselling sessions	Significant difference in: – participant–rated homework completion 2.22 (0.73) vs. 1.96 (0.55), *p* = .006	No significant difference in improvement of anxiety symptoms
Raue et al., 2019	USA	Cluster RCT	24 202	Primary care physicians Racial and ethnic minority primary care patients (65+) with a depressive disorder	Psychotherapy	Shared decision–making intervention aligning treatment decisions with patient preferences and values through: (i) psychoeducation on depression, treatment options and expected outcomes and (ii) addressing psychological and practical barriers to engagement like motivation, stigma, cost or transportation.	Treatment as usual	No effect on number of psychotherapy sessions attended	No significant difference in improvement of depression symptoms
Reis and Brown, 2006	USA	RCT, 4 arms	125	Primary care patients seeking treatment for an adjustment, mood or anxiety disorder	Psychotherapy	Arm 1 watched 12–min training video introducing patient to psychotherapy with clinical vignettes of desired in–therapy behaviours Arm 2 watched pretherapy video unrelated to psychotherapy, discussed and preliminarily agreed upon an estimated number of sessions with therapist based on patient problems and goals Arm 3 watched the psychotherapy training video and preliminarily agreed upon an estimated number of sessions with therapist	Pretherapy video unrelated to psychotherapy with no	Significant reduction in dropout score (defined as judgment of clinical progress at time of two missed sessions or one missed session without rescheduling) for intervention vs. control group: (M–28.85) vs. (M–33.36), *p* < .001	Not reported
Stein et al., 2020	USA	RCT	100	College students with depression	Single–session Brief Behavioural Activation Treatment for Depression (BATD)	Practiced an activity that student identified in BATD as challenging yet rewarding (such as calling a friend or going on a walk) with immediate debrief on thoughts, feelings, and strategies for completing the activity at home	Only BATD	Significant increase in activity completion 4.9 (1.53) vs. 4.26 (1.26), *p* = .047	No significant difference in improvement of depression symptoms
Wang et al., 2022	China	Cluster RCT	20 245	Communities Older adults (60–90 years) with post stroke depression	CBT	The intensive health education consisted of the following: health education, special lectures and home visits	Routine community treatment	Significantly higher compliance rates of medical and psychotherapeutic treatments in the experimental group compared to control 6.03% vs. 22.2, *p* < .01	Not reported
Wells *et al.*, 2020	United Kingdom	nRCT	51	Cancer patients with depression/anxiety referred to a psycho–oncology service	Mindfulness–cognitive therapy (MBCT)	Smart–messaging text intervention including reinforcement of therapy content, homework reminders and therapy reminders	Only MBCT	Significant difference in rate of therapy completion (defined as attending at least 5 of 8 sessions) 87% vs. 38%, *p* = .007	Significant improvement in depression symptoms *B* =–2.33 (reduced by 2.33 points more among intervention group) 95% CI: (.76, 3.89), *p* = .004
Westra and Dozois, 2006	Canada	RCT	55	Publicly insured patients with anxiety disorder referred to an anxiety and affective disorders clinic	CBT	Motivational interviewing pre–treatment focused on ambivalence, motivation and obstacles to anxiety change	Only CBT	Significant difference in patient–rated homework compliance 4.28 (0.49) vs. 3.79 (0.52), *p* < .05	Significant difference in improvement of anxiety symptoms between arms: mean *Z* score change = 2.57 (1.47) vs. 1.27 (1.27), *p* < .05
Westra et al., 2009	Canada	RCT	76	Community members with generalised anxiety disorder (GAD)	CBT	Motivational interviewing pre–treatment focused on ambivalence, motivation and obstacles to anxiety change	Only CBT	Significant increase in therapist–rated homework compliance 4.59 (0.53) vs. 4.19 (0.80), *p* = .013	No significant difference in improvement of anxiety scores or functional impairment

### Participant characteristics

The mean age of participants across the included studies ranged from 19.6 to 69 years. Depressive disorders (*n* = 9) were identified based on one of the following: Patient Health Questionnaire (PHQ-9), Hamilton Depression Rating Scale, Beck Depression Inventory and clinician diagnosis. Anxiety disorders (*n* = 6) included social anxiety disorder, panic disorder and generalised anxiety disorder, and were identified using one of the following: Anxiety Sensitivity Index (ASI), Generalised Anxiety Disorder-7, Social Interaction Anxiety Scale, State-Trait Anxiety Inventory and clinician diagnosis. Seven studies enrolled participants who had an adjustment/mood disorder, or a CMD comorbid with another condition (substance use, cancer). The oldest study, conducted in 1964, described its participants as having a ‘psychoneurotic disorder’ (Hoehn-Saric et al., 1964).

The following section describes the various interventions that were tested to improve adherence to psychological treatments for CMDs.

### Interventions

Seven studies involved reminders (Clough, 2014; Delgadillo et al., 2015), content reinforcement between sessions (Furber et al., 2014; Alfonsson et al., 2019; Pérez et al., 2021) or a combination of both (Aguilera et al., 2017; Wells et al., 2020). Five studies (Daley et al., 1998; Westra and Dozois, 2006; Westra et al., 2009; Barrera et al., 2016; Peters et al., 2019) tested interventions involving principles of motivational communication between therapist and patient. Six studies (Hoehn-Saric et al., 1964; Latour and Cappeliez, 1994; Reis and Brown, 2006; Delgadillo and Groom, 2017; Stein et al., 2020; Wang et al., 2022) tested interventions consisting of pre-therapy orientation to set shared expectations and familiarise patients to the therapy. Two studies (Miranda et al., 2003; Raue et al., 2019) implemented clinical case management services to address structural barriers to engaging in therapy for depression. One study (Mohr et al., 2012) aimed to reduce practical challenges to therapy adherence by employing telephone-based delivery.

Two studies (Avishai et al., 2018; Jurinec and Schienle, 2020) incorporated unique psychological principles in self-delivered interventions targeting adherence to therapy and its components. One of these studies administered a placebo to the intervention arm, alongside a psychoeducation course to enhance motivation for homework completion (Jurinec and Schienle, 2020). The other study involved implementation intentions to sustain attendance at a group psychoeducation programme (Avishai et al., 2018).

All except five interventions (Latour and Cappeliez, 1994; Furber et al., 2014; Delgadillo et al., 2015; Raue et al., 2019; Pérez et al., 2021) were effective in improving at least one adherence outcome. Sixteen studies also reported a clinical outcome or a measure of well-being. Six studies were effective in improving at least one clinical outcome (Hoehn-Saric et al., 1964; Miranda et al., 2003; Westra and Dozois, 2006; Delgadillo and Groom, 2017; Jurinec and Schienle, 2020; Wells et al., 2020) and three studies were effective in improving at least one measure of well-being or disability (Hoehn-Saric et al., 1964; Daley et al., 1998; Miranda et al., 2003).

All the interventions and their effect on adherence outcomes are discussed in detail under relevant categories below and presented in Supplementary Material, Appendix 2.

#### Reminders and between-session engagement

Seven studies utilised an automated text-messaging software to deliver reminders (Clough, 2014; Furber et al., 2014; Delgadillo et al., 2015; Aguilera et al., 2017; Alfonsson et al., 2019; Wells et al., 2020; Pérez et al., 2021) to patients’ mobile phones at times and dates relevant to their therapy sessions. This included a single text message reminder prior to the first therapy appointment (Delgadillo and Groom, 2017) or multiple texts throughout the week, reminding participants to attend therapy as well as regularly practice cognitive exercises such as meditation taught during therapy (Aguilera et al., 2017; Wells et al., 2020). In addition to reminders, these interventions incorporated text messages that reinforced content taught in therapy. These messages were tailored in collaboration with patients (Furber et al., 2014; Aguilera et al., 2017; Alfonsson et al., 2019) or were interactive (Furber et al., 2014). One study evaluated the effectiveness of a blended treatment approach as compared to treatment as usual (TAU) alone (Pérez et al., 2021). The blended approach comprised of the TAU alongside an internet intervention that ensured between-session engagement by providing psycho-educational information, supportive monitoring and regular feedback (Pérez et al., 2021).

Out of the seven studies, four reported the intervention being significantly superior to the control in at least one adherence measure: homework completion (Alfonsson et al., 2019), duration in treatment (Aguilera et al., 2017), therapy completion rate (Wells et al., 2020) or reduction in dropouts (Clough, 2014). Out of the four studies that reported symptomatic/functional outcomes (Furber et al., 2014; Aguilera et al., 2017; Wells et al., 2020; Pérez et al., 2021) only one study reported a statistically significant difference of a clinical outcome (depression symptoms) between trial arms (Wells et al., 2020).

## Motivational interviewing

Five studies (Daley et al., 1998;Westra and Dozois, 2006; Westra et al., 2009; Barrera et al., 2016; Peters et al., 2019) tested interventions involving principles of motivational interviewing (MI), a guided counselling style that elicits behavioural change in patients by exploring and resolving ambivalence. Four studies (Westra and Dozois, 2006;Westra et al., 2009; Barrera et al., 2016; Peters et al., 2019) employed MI methods that were adapted for therapy ‘pre-treatment’ by Westra and Dozois (2003). The pre-treatment was delivered individually by graduate-level clinical psychologists prior to the onset of therapy and ranged from a single pre-treatment session (Reis and Brown, 2006) to four weekly pre-treatment sessions (Stein et al., 2020). One study utilised a different approach to motivational counselling as described by Hester and Miller (2003), for substance use disorders and incorporated these principles into the therapy sessions (Hoehn-Saric et al., 1964).

Compared to the control, MI pre-treatments were found to significantly improve the number of sessions attended (Daley et al., 1998; Barrera et al., 2016), client-rated and therapist-rated homework compliance (Westra and Dozois, 2006; Peters et al., 2019) and rate of treatment completion (Daley et al., 1998; Westra and Dozois, 2006). Three of the studies reported no significant differences in improvement of anxiety symptoms between arms (Westra et al., 2009; Barrera et al., 2016; Peters et al., 2019). One study reported significant improvement in anxiety symptoms in the intervention arm compared with the control (Westra and Dozois, 2006), while another reported reduced rehospitalisations and time spent in in-patient treatment (Daley et al., 1998).

### Therapy orientation

Six studies (Hoehn-Saric et al., 1964; Latour and Cappeliez, 1994; Reis and Brown, 2006; Delgadillo and Groom, 2017;Stein et al., 2020; Wang et al., 2022) tested interventions to set shared expectations, orient patients to the components of therapy, and practice desirable therapy roles and behaviours in a safe and less personal environment. These interventions were delivered to individuals (Hoehn-Saric et al., 1964; Reis and Brown, 2006; Delgadillo and Groom, 2017; Stein et al., 2020; Wang et al., 2022) or groups (Latour and Cappeliez, 1994; Delgadillo and Groom, 2017) through a single session (Hoehn-Saric et al., 1964; Stein et al., 2020) or multiple sessions (Latour and Cappeliez, 1994; Reis and Brown, 2006; Delgadillo and Groom, 2017; Wang et al., 2022). Each of these interventions combined various components of pre-therapy education, expectation-setting and role-playing. Delivery formats were multimodal: videos showcasing scenarios that mimic desired therapy behaviours (Latour and Cappeliez, 1994; Reis and Brown, 2006), and in-person sessions to learn and practice exercises in completing homework (Stein et al., 2020). Expectation-setting had to do with the role of patient and therapist in the therapeutic relationship (Ogrodniczuk *et al.*, 2005; Wang et al., 2022), anticipated difficulties to adherence (Stein et al., 2020; Wang et al., 2022) and time taken by the therapy to take effect (Hoehn-Saric et al., 1964; Reis and Brown, 2006; Wang et al., 2022).

Five out of six interventions demonstrated significant improvements in adherence outcomes compared to controls, such as higher homework completion (Stein et al., 2020), longer treatment duration (Delgadillo and Groom, 2017), fewer dropouts (Reis and Brown, 2006) and increased attendance at sessions (Hoehn-Saric et al., 1964; Delgadillo and Groom, 2017; Wang et al., 2022). Out of the three studies that reported clinical and well-being outcomes (Hoehn-Saric et al., 1964; Delgadillo and Groom, 2017; Stein et al., 2020), one study reported significant differences in improvement of anxiety symptoms between arms (Delgadillo and Groom, 2017). Another study reported significant differences in patient-reported symptom improvement and therapist-rated global improvement between arms (Hoehn-Saric et al., 1964).

### Case management services

Two interventions (Miranda et al., 2003; Raue et al., 2019) attempted to increase adherence to therapy through case management with support in overcoming structural barriers. Patient-centred case management services that assisted older adults (engaging in treatment decisions, scheduling appointments and facilitating transportation) were superior to controls in getting patients to start therapy, but they did not retain them in therapy any better than controls (Raue et al., 2019). Another study which involved clinical case management that supported patients in resolving self-reported problems related to housing, employment and interpersonal relationships, helped improve session attendance and reduce dropout rates in a sub-sample of individuals from low socioeconomic backgrounds (Miranda et al., 2003). It also reported significant differences in improvement of depression symptoms and social functioning between arms (Miranda et al., 2003).

### Telephone-based therapy

One intervention sought to address practical barriers to engaging in therapy for depression in a clinical setting (Mohr et al., 2012). It tested telephone-based therapy against face-to-face CBT and found significant improvements in number of sessions attended and reduced dropout rates. However, significantly greater improvement in clinical outcomes was seen in face-to-face CBT as opposed to telephone-based therapy.

### Placebo intervention

One study administered a placebo alongside a CBT-based outpatient psychoeducation course to enhance motivation for homework completion (Jurinec and Schienle, 2020). It was a blue glass bottle and a dropper with 30 ml sunflower oil, labelled ‘golden root oil’ (rhodiola rosea). This placebo label was chosen because rhodiola rosea was widely used as a traditional medicine for several symptoms and disorders (including depression). Intervention group participants were instructed to consume three drops (0.15 ml) of the placebo oil 10 min before the daily relaxation exercise advised in the outpatient course. The intervention was superior to control in increasing homework completion and led to a significantly greater improvement in depression symptoms (Jurinec and Schienle, 2020).

### Implementation intention

One study involved implementation intentions which are brief, simple, low-cost, self-regulatory strategies in the form of an “if-then plan” (Avishai et al., 2018). These involved anticipating barriers and creating mitigation strategies, to sustain attendance at a group psychoeducation programme. The intervention intended to help patients in downregulating feelings of concern associated with attending large group sessions. It was superior to the control in significantly increasing session attendance and treatment duration.

### Delivery of interventions

#### Mode and setting

Eleven of the interventions were delivered in-person; mainly in university psychiatric clinics (*n* =5), primary healthcare centres (*n* = 4) or community health centres (*n* = 2). Seven of the interventions employed automated text messages. Others involved telephonic delivery (*n* = 2), a pre-programmed website (*n* = 1) and delivery via mail (*n* = 1). One intervention consisting of clinical case management, provided services both in-person at an outpatient mental healthcare centre and over telephone.

#### Delivery agent

Seven interventions, involving MI and therapy orientation, were delivered by clinical psychologists and doctoral graduate students of psychology. Seven interventions did not involve a delivery agent and relied on computer programmes sending automated text messages to participants or pre-programmed websites doing the same. Other interventions were delivered by a range of practitioners such as neurologists (*n* = 1), psychiatrists (*n* = 1), registered nurses (*n* = 1) and researchers with a post-graduate degree (*n* = 1). Two interventions were self-delivered (Avishai et al., 2018; Jurinec and Schienle, 2020) and two studies did not specify the characteristics of the delivery agent (Daley et al., 1998; Miranda et al., 2003). One intervention was delivered by both licensed social workers with master’s degrees and doctoral graduate students of psychology (Reis and Brown, 2006).

### Implementation outcomes

Five studies reported implementation outcomes. All five studies reported on acceptability of the intervention (Furber et al., 2014; Delgadillo and Groom, 2017; Alfonsson et al., 2019; Jurinec and Schienle, 2020; Wells et al., 2020), defined as the perception among stakeholders that a given treatment or service was agreeable or satisfactory (Proctor et al., 2009). Four of the studies based their findings on perspectives of the participants and one explored perspectives of therapists on patient acceptability of the intervention (Furber et al., 2014). Three of them explored patient perspectives using Likert scales (Delgadillo and Groom, 2017; Alfonsson et al., 2019; Jurinec and Schienle, 2020) and two conducted qualitative interviews with therapists (Furber et al., 2014) and participants (Wells et al., 2020).

In one study (Alfonsson et al., 2019), participants rated their perception of the text messages in terms of helpfulness, annoyance, redundancy and value on a scale ranging from 0 (not at all) to 4 (absolutely). The threshold for being “satisfied with the intervention” was considered a score of 50%. Four participants rated the text messages at 50% or above, indicating satisfaction, while three participants rated them as neutral or irrelevant (scoring below 50%). In the open feedback section, participants expressed that the text messages served as helpful reminders for exercises and helped keep the treatment on track between sessions. However, they also felt pressured when unable to complete all assignments and found the text messages lacking personalisation. Another study employed a similar approach, where participants evaluated the effectiveness of a placebo on a 10-point Likert scale, with 10 representing extreme effectiveness. The mean score was 8.19 (SD = 1.15) (Jurinec & Schienle, 2020).

In another study (Delgadillo and Groom, 2017), a brief acceptability questionnaire was designed for the intervention, consisting of 3-point Likert-scale items rated from 0 to 10 (high). Patients rated each session based on relevance, the quality of the presentation and quality of the materials. The average ratings were combined to form a single measure of acceptability, which was then compared across different diagnostic groups. The results showed no significant differences across the diagnostic groups.

In one of the studies (Furber et al., 2014), therapists reported that patients initially found the messages useful in therapy but perceived them as less useful over time. In another study (Wells et al., 2020) qualitative interviews were conducted with patients who either declined or accepted smart messaging (*n* = 15). Among those who declined text messaging in the control group (*n* = 2), the main reason given was a lack of confidence in using mobile phones. On the other hand, participants who accepted messaging in the intervention group viewed it as a helpful reminder, motivating them to complete exercises and maintain mindfulness practices in their daily lives (Wells et al., 2020). Interviews also revealed a secondary theme of feeling a personal connection, despite being aware that the texts were automated.

### Risk of bias

The majority of RCTs assessed using the RoB 2 tool were of high quality, but a third of the studies (*n* = 6) demonstrated moderate or high (Westra et al., 2009; Peters et al., 2019; Raue et al., 2019; Stein et al., 2020; Wang et al., 2022) risk of bias. The most common domain of concern was in the selection of the reported result (Clough, 2014; Barrera et al., 2016; Avishai et al., 2018; Stein et al., 2020; Pérez et al., 2021; Wang et al., 2022). Some RCTs also demonstrated a moderate or high level of bias in the following domains: allocation concealment or randomisation (Reis and Brown, 2006; Wang et al., 2022), deviations from intended interventions (Westra et al., 2009; Peters et al., 2019), outcome measurement (Reis and Brown, 2006; Raue et al., 2019) and missing outcome data (Stein et al., 2020).

While the nRCTs included in the review were generally of high quality, some studies (Furber et al., 2014; Aguilera et al., 2017; Wells et al., 2020) had moderate risk of bias due to confounding. One study (Daley et al., 1998) lacked enough information to be assessed for bias due to confounding, deviations from intended interventions and selection of reported results. The risk of bias assessments of all studies can be found in Supplementary Material, Appendix 3.

## Discussion

We conducted this systematic review to examine interventions designed to improve patient adherence to psychological treatment for CMDs. We identified eight groups of strategies that researchers have employed across various settings to improve adherence to psychological treatment. The heterogeneity of how adherence outcomes are defined and measured, as well as the diversity in patient population, selection methods, follow-up periods, content and delivery of interventions tested, prevent us from drawing comparative conclusions about the effectiveness of specific types of adherence interventions. However, while there are not sufficient similarities among these findings to warrant statistical pooling of the results, it is important to note the trends of the different interventions. For instance, 9 out of 10 studies (Hoehn-Saric et al., 1964; Daley et al., 1998; Reis and Brown, 2006; Westra and Dozois, 2006; Westra et al., 2009; Barrera et al., 2016; Delgadillo and Groom, 2017; Peters et al., 2019; Stein et al., 2020) that provided some form of preparation to patients on the practical and theoretical aspects of therapy participation were found to be effective in improving at least one adherence measure. These preparation strategies included MI, role and relationship induction and practical expectations surrounding therapy.

Therapy orientation process has been previously described as an opportunity for patients to acquire information, observe and participate in experiences and then develop skills that link the information and experience in the mind of the patient (Piper et al., 1999). Research shows that this information allows patients to set realistic expectations and evokes feelings of satisfaction and hope when those expectations are met in the process of therapy (Hoehn-Saric et al., 1964). Additionally, it reduces the apprehension and anxiety around therapy, which may otherwise lead to counterproductive behavioural patterns like avoidance or resistance (France and Dugo, 1985).

Similarly, after employing MI style communication between therapist and patient, Barrera and colleagues saw improvement in motivation to change (Barrera et al., 2016), which is necessary for action-oriented treatments such as CBT. By linking provided information with an experience, a patient might be able to change the thinking that interrupts the connection of knowledge (“I *s*hould attend therapy”, “therapy will help me”) with action (attending therapy).

Reminder-based interventions may play a different role in improving adherence. Older studies have suggested that appointment reminders (in the form of letters and phone calls) can reduce therapy dropout rates (Hochstadt and Trybula, 1980; Sherman and Anderson, 1987; Swenson and Pekarik, 1988) but studies on text messaging are still relatively new. This review suggested that, in combination with supportive monitoring and content reinforcement messages, these interventions provide a personal connection that makes patients feel supported and motivated, strengthen the therapeutic alliance and provide longitudinal engagement that increases the likelihood that a person will return to therapy even after a period of absence.

Case Study 1: **Automated text messages**

In the US-based study by Aguilera et al. (2017), five types of automated text messages were sent in Spanish: (a) daily mood rating prompt (e.g., “What is your mood right now on a scale of 1 to 9?”); (b) Feedback based on mood ratings (e.g., “Treat yourself with kindness, the same way you would treat a loved one”); (c) daily message reinforcing live therapy content (e.g., “Do at least one new pleasant activity this week”); (d) weekly reminder to attend psychotherapy sent the night before session (e.g., “Are you coming to group this week?”); (e) a monthly opt-out message to terminate message delivery, if desired (e.g., “To stop receiving these messages, respond with the word STOP”). Text messages were delivered multiple times a day, and the patients were allowed to customise the time of day to receive reminders. At the start of each therapy session, patient’ mood data were displayed on a whiteboard to evaluate their mood states over the past week and apply therapeutic tools to specific events. This intervention was administered for the full duration of the 16-week manualised group CBT at a behavioural health outpatient clinic in an urban public hospital, targeting a sample of low-income Spanish-speaking Latino patients.

Given that attendance is necessary for in-person therapy, any intervention that improves session attendance should be critical for therapy outcome (Hansen et al., 2002; Robinson et al., 2020). While Robinson and colleagues established a curvilinear relationship between number of sessions and response to treatment from naturalistic studies, our synthesised findings from clinical trials do not necessarily corroborate this idea. Out of the eight studies that found a significant improvement in session attendance (Hoehn-Saric et al., 1964; Daley et al., 1998; Miranda et al., 2003; Mohr et al., 2012; Barrera et al., 2016; Delgadillo and Groom, 2017; Avishai et al., 2018; Wang et al., 2022), only three found a correlating and significant improvement in clinical outcomes (Hoehn-Saric et al., 1964; Miranda et al., 2003; Delgadillo and Groom, 2017). This may reflect the possibility of a more nuanced link between treatment dosage and treatment outcomes.

Case Study 2: **Motivational interviewing**

One study conducted in the United States (Daley et al., 1998) integrated dual disorder recovery counselling and motivational therapy. This integrated treatment strategy targeted both substance use and common mental health disorders, aiming to equip patients with the skills to foresee and manage challenges that hinder adherence by leveraging their own internal resources for change. The intervention employed the FRAMES acronym, which stands for: providing **F**eedback on substance use and mood problems, emphasising the patient’s **R**esponsibility for change, offering **A**dvice on how to change, presenting a **M**enu of change options, showing Empathy towards the patient and fostering **S**elf-efficacy or confidence in their ability to change. The study applied these principles to individuals who were receiving outpatient care for depression and cocaine dependence after being discharged from a university psychiatric hospital’s dual diagnosis in-patient unit, during individual and group CBT sessions over 4 weeks.

However, it is difficult to make any such assertion without a consistent definition of adherence among researchers and clinicians. There is also a lack of a robust research base with large prospective studies capturing the effect of adherence interventions on both adherence outcomes and clinical outcomes. In addition, frequently assessing patient perceptions (their expectations, motivations and progress) throughout treatment may further illuminate why patients continue to attend therapy- and perceive-related benefits.

It is important to note that all the studies found in this systematic review tested interventions dealing with patient-related factors of adherence. These interventions are critical for working within a patient’s ability to access internal resources of knowledge, skills and beliefs that engage or deter a patient from therapy. However, it is misleading to believe that patients are solely responsible for adherence to therapy. An evidence base that neglects the other factors affecting a person’s capacity to adhere to psychological treatment will only partially resolve the issue of poor session attendance and completion rates (Sabaté, 2003). Relatively less research has been conducted globally on the other dimensions of adherence as defined by WHO’s framework on interacting dimensions affecting adherence (Sabaté, 2003), including factors related to the social and economic status of the patient, therapist characteristics, the severity and progression of the patient’s condition, presence of comorbidities and the complexity and duration of therapy itself (Peh et al., 2021).

The benefit of intervening in other dimensions can be seen in a limited number of studies included in this review (all conducted in United States). For example, Mohr *et al.* demonstrated the early improvement in session attendance and dropout rate by switching from in-person to telephone-based therapy (Mohr et al., 2012). This intervention reduces the logistical and motivational barriers to physical attendance at a clinic. Miranda *et al.*’s case management approach improved session attendance and dropout rates (Miranda et al., 2003). It worked on social- and economic-related dimensions by addressing other areas of difficulty (housing, employment, relationships) that could interfere with or disrupt consistent treatment. Several such strategies working in parallel and targeting multiple dimensions of adherence may work to improve adherence to psychological treatment (Raue et al., 2019).

Case Study 3: **Therapy orientation**

One study from the United Kingdom used pre-treatment lecture style seminars (Delgadillo and Groom, 2017) that were delivered in a group setting by CBT practitioners, to patients diagnosed with anxiety or depressive disorders. The seminar content included: (a) clarification on the roles of the patient and therapist; (b) psychoeducation on how transdiagnostic processes (thinking, behaviour, attention and memory) contribute to psychological distress and (c) an overview of generic aspects of CBT and common misconceptions related to it. Each seminar revolved around one central theme; such as problematic thinking processes, behavioural change and emotion regulation. The seminars used accessible language and materials (slides, booklets and videos) along with exercises that emphasised personal experiences and self-reflection. Participants had the opportunity to ask questions and engage in brief discussions about the content. The three weekly seminars of 90 min were followed by 20 sessions of individual CBT.

It is noteworthy to mention that all studies included in this review were conducted in high-income or upper-middle-income countries. The lack of studies testing the included adherence strategies in LMIC contexts limits the generalisability of our findings, especially when differences in health systems and social and economic factors in LMICs may influence how similar interventions will exert their effects. Additionally, the inclusion of only two studies with minority populations (Aguilera et al., 2017; Avishai et al., 2018) limits drawing conclusions about the applicability of our findings to diverse contexts and populations. Recent advances in non-specialist-delivered psychosocial treatments and task sharing in LMICs (Singla et al., 2017) could mean “adherence to treatment” would look significantly different from the strategies reported in this review since all the included studies had specialists as delivery agents. Adherence strategies applied for other chronic illness treatments (e.g., tuberculosis, cardiovascular diseases) in LMICs (Pradipta et al., 2020; Loots et al., 2021; Ogungbe et al., 2021) emphasise home-based, group-based or peer-led approaches, such as home visits by community agents and peer-support groups to enhance adherence. Other reviews focusing on adherence in LMIC contexts (Pradipta et al., 2020; Loots et al., 2021; Boima et al., 2024) also emphasise multicomponent interventions that integrate treatment initiation with adherence strategies (e.g., patient education and home visits by healthcare workers), social with psychological interventions (e.g., monetary incentives with MI) or digital with in-person interventions (e.g., SMS reminders and nurse follow-up).”

Evidence also suggests that multi-level interventions, such as combining individual therapies with community-based or facility-based programmes, can be effective in LMICs (Reif et al., 2020; Pugh et al., 2022). Therapy orientation and content reminders, which have proven effective in HIC settings, also show promise in LMIC settings when combined with additional support such as case management, which targets non-patient-related barriers (structural challenges of clinic-based care, and poor provider relationships). Innovative group-based approaches like “adherence clubs” offer valuable opportunities to provide comprehensive services, including education and information on additional support resources like addressing food insecurity. However, further research is needed to tailor these interventions to address the complex, non-patient-centric aspects of treatment adherence, ensuring they cater to the specific needs of the target population and context.

Further research is needed to address the methodological limitations of currently published findings, as well as the conceptual limitations surrounding the definition and measure of adherence. We hope this review highlights the gap in intervention studies targeting social, economic, health system, healthcare team and therapeutic factors as the causes of problems with adherence to psychological treatment. A uniform yet inclusive understanding and measure of adherence to psychological treatment can serve practical purposes for research and policymaking and will maximise the benefit of therapy for patients in clinical settings.

This review further adds to the existing literature base on adherence to psychological treatments, a subject about which there is considerable discussion but little consensus or progress. To our knowledge, only one prior systematic review (Oldham et al., 2012) has assessed the literature for interventions that improve adherence to psychological treatment. We believe that our review has relative strengths compared to the previous review in terms of choosing a broader definition of adherence, searching a higher number of databases for studies, including a wider range of studies, and acknowledging the different approaches of adherence needed for CMDs.

There are a number of limitations to our findings and review process. A broad conceptualisation of adherence inevitably means procuring a heterogeneity of outcome measures, definitions of those measures, follow-up periods, delivery methods, intervention content, intervention duration and psychological treatments itself. While we believe this proved useful in illustrating the landscape of research currently in existence on the topic, we recognise this does not provide sufficient consistency for meta-analysis or definitive conclusions on the “most effective” strategy. We did not search grey literature, publications in languages other than English, and regional databases, which may bias our results and could have excluded studies conducted in LMICs. The quality assessment of the studies did not impact the weight given to them in the narrative synthesis. Finally, the sample sizes across studies varied significantly, with some included studies having as few as seven and eight individuals, which decreases the precision and generalisability of the results (Avishai et al., 2018; Alfonsson et al., 2019).

We hope that our review will orient researchers, policymakers, clinic administrators and care providers to the array of interventions that have been tested to improve psychological treatment adherence for CMDs. Integrating adherence strategies in mental health services is crucial for enhancing treatment effectiveness and patient outcomes for individuals with CMDs. By incorporating these strategies into treatment services, therapists can better engage and support their patients, leading to potentially better mental health outcomes and overall well-being.

## Supporting information

Biswal et al. supplementary material 1 Biswal et al. supplementary material

Biswal et al. supplementary material 2 Biswal et al. supplementary material

Biswal et al. supplementary material 3 Biswal et al. supplementary material

Biswal et al. supplementary material 4 Biswal et al. supplementary material

Biswal et al. supplementary material 5 Biswal et al. supplementary material

## Supplementary material

The supplementary material for this article can be found at http://doi.org/10.1017/gmh.2024.94.
