# Supplementary material for: Interventions for improving adherence to psychological treatments for common mental disorders: a systematic review
Source: Glob Ment Health (Camb). 2024 Oct 17;11:e83. doi: 10.1017/gmh.2024.94 (PMC11504929; doi:10.1017/gmh.2024.94)
Supplement: Biswal et al. supplementary material 1 — Biswal et al. supplementary material [file S2054425124000943sup001.pdf]

Citation

Simone Renault, Ratika Bhandari, Abhijit Nadkarni, Ankur Garg, Vikram Patel, Urvita Bhatia, Miriam Sequeira. Interventions for improving adherence to psychological treatment for common mental disorders: a systematic review. PROSPERO 2021 CRD42021266680 Available from: https://www.crd.york.ac.uk/prospero/display_record.php?ID=CRD42021266680

Review question

Which interventions have been tested for improving adherence to psychological treatment for common mental disorders?

Searches

Peer reviewed literature from the following databases:
MEDLINE
PsycINFO
Embase

Global Health

CINAHL

CENTRAL

Search dates: 12/07/2021 - 16/07/2021

The review will be restricted to studies published in English.

Searches will be conducted based on outlined search strategy.

Types of study to be included

Inclusion:

Randomized controlled trials
Non-randomized controlled trials

Exclusion:

Quasi-experimental studies

Observational studies

Case studies

Case series

Reviews

Meta-analyses

Commentaries

Condition or domain being studied

Common mental disorders such as depression, anxiety disorders, and somatoform disorders

Participants/population

Inclusion: Study population Persons 18 years and older* experiencing one or more of the following common mental disorders:

- Depressive disorder

- Common anxiety disorders including generalized anxiety disorder, panic disorder, social anxiety disorder, phobias, or anxiety disorder not otherwise specified

- Somatoform disorders including somatic symptom disorder, conversion disorder, and illness anxiety disorder

- Neurotic disorders

- Common mental disorders

Persons with the above conditions will be eligible if they have been identified through one or more of the following: 1) ICD or DSM criteria; 2) positive screen by a standardized diagnostic instrument (e.g. PHQ-9, GAD-7); or 3) clinician diagnosis.

Participants may experience any of the above conditions in isolation or in conjunction with another physical or mental health condition.

Studies that include participants with a mix of mental health conditions will be included if ?80% of participants meet our eligibility criteria.

*Mean age of participants must be 18 years or older.

Intervention(s), exposure(s)

Inclusion: Interventions designed and/or tested to directly target patient adherence (as indicated by study authors or primary outcome) to an evidence-based individual or group psychological treatment* such as cognitive behavior therapy, problem-solving therapy, or psychodynamic psychotherapy

*Psychological treatment may be delivered by itself or in combination with pharmacological or psychosocial treatment in-person or via telecommunication technologies (e.g., tele-counselling)

Exclusion: Interventions not designed/tested to directly target adherence but which lead to improved adherence as an indirect/secondary outcome

Interventions designed/tested to target adherence to web- or mobile app-based psychological treatments

Interventions designed/tested to exclusively target adherence to pharmacological treatment

Comparator(s)/control

Inclusion:

Treatment as usual/usual care
Enhanced usual care

Another adherence intervention

Delayed intervention

Wait-list control

Context

All settings globally

Main outcome(s)

Inclusion: Objective measures of adherence to psychological treatment as defined by the extent to which a person continues with or completes psychological treatment in correspondence with agreed recommendations from a mental health care provider.

Outcomes for adherence to psychological treatment will be measured starting after the completion of an initial session of psychological treatment and continue until the completion of a final session of psychological treatment, defined by either the fixed endpoint of a treatment protocol or by mutually agreed discharge between service user and provider.

This may include, but is not limited to:

• Appointment attendance

• Homework compliance

• Treatment uptake, engagement, motivation, utilization, participation, or retention

• Treatment discontinuation, dropout, withdrawal, lost to follow-up, attrition, or premature termination

• Treatment fidelity

• Treatment interruption

• Treatment completion or discharge

Exclusion: Measures related to initiation of psychological treatment (i.e., attendance at initial therapy appointment)

Measures of effect

Risk ratio, effect size, standardized mean difference

Additional outcome(s)

Content and delivery methods of identified interventions

Clinical outcomes related to mental health symptoms, wellbeing, disability, or quality of life

Acceptability and feasibility of identified interventions, where available

Measures of effect

Risk ratio, effect size, standardized mean difference

Data extraction (selection and coding)

1. Search will be followed by merging & importing all results from the defined databases into Covidence.

2. Remove duplicate records of the same report.

3. Two reviewers will screen all title and abstracts; justification for exclusion will be recorded in Covidence. Disagreements between the two reviewers will be resolved by a third reviewer. Full-texts of the remaining, potentially relevant reports will be retrieved.

4. Multiple reports of the same study will be linked together. Studies on the same intervention conducted in

different contexts will be included as separate studies. Multiple studies on the same intervention with the same context and outcomes but published at different times, we will include the most recent study.

5. Full-text of each study will be reviewed independently by two reviewers. Justification for exclusion will be recorded in Covidence. Disagreements between reviewers will be resolved by a third reviewer.

Excel data extraction form will be made for the review with key findings relevant to the study objectives, pre-piloted, and utilized following full-text screening of eligible studies.

Process and results of assessing eligibility and data extraction will be presented in a flowchart following the Preferred Reporting Items for Systematic Reviews and Meta-Analyses (PRISMA) guidelines. Attempts will be made to collect any missing information from study authors via email.

Data extraction categories include: Title; Author; Year of publication; Year of trial

- Trial country

- Aims & objectives

- Trial design

- Trial setting (community-based, facility-based)

- Sample size

- Participant characteristics (age, gender, diagnosis, comorbidities)

- Psychological treatment characteristics (type of evidence-based treatment, stand-alone vs. paired with pharmacologic treatment, delivery method of treatment)

- Type of adherence intervention (tech-based, education-based)

- Delivery method of adherence intervention

- Content/components of adherence intervention

- Type of control/comparison intervention

- Follow-up period

- Adherence outcome measures

- Adherence outcome results

- Clinical outcome measures and results (mental health symptoms, wellbeing, disability, quality of life)

- Implementation outcome measures and results (acceptability, feasibility)

Risk of bias (quality) assessment

For studies that are deemed eligible for inclusion, a quality assessment will be conducted using: Cochrane Risk of Bias Version 2 (RoB2) tool for randomized trials and Cochrane ROBINS-I tool for nonrandomized trials

Using these tools, two reviewers will assess the quality of included papers. Assessments will be compared, and any disagreements will be resolved by a third reviewer.

Strategy for data synthesis

A descriptive analysis of the data will be undertaken to synthesize findings associated with each study

objective. Results on the content and delivery method of included interventions will be reported in a narrative format, accompanied by tables to convey relevant study components.

If the data allows for meta-analysis, we will compare the primary outcome and clinical outcomes (if available) between arms. We will synthesize the risk ratio for the primary outcome (with 95% confidence intervals), effect size, and a standardized mean difference between arms for each trial. Publication bias will be assessed using funnel plots.

Where possible, we will provide a narrative synthesis on the acceptability of interventions and feasibility of their delivery.

Analysis of subgroups or subsets

If there are a sufficient number of trials, we will conduct subgroup analyses for studies conducted in low- and middle-income vs. high-income countries, length of follow-up period (short vs. long), and delivery method of intervention (tech-based vs. not), reporting on the effect size and standardized mean difference for each.

Contact details for further information

Simone Renault
Simone.Renault1@student.lshtm.ac.uk

Organisational affiliation of the review

London School of Hygiene & Tropical Medicine
King's College London
Sangath, India
https://www.lshtm.ac.uk/

https://www.kcl.ac.uk/

https://sangath.in/

Review team members and their organisational affiliations

Miss Simone Renault. London School of Hygiene & Tropical Medicine
Ms Ratika Bhandari. Sangath
Dr Abhijit Nadkarni. Sangath and London School of Hygiene & Tropical Medicine
Mr Ankur Garg. Sangath
Dr Vikram Patel. Sangath and Harvard T.H. Chan School of Public Health
Ms Urvita Bhatia. Sangath and Oxford Brookes University
Ms Miriam Sequeira. Sangath

Type and method of review

Systematic review

Anticipated or actual start date

12 July 2021

Anticipated completion date

01 September 2021

Funding sources/sponsors

NIMH fund granted to Sangath [Grant number: 1U01MH115504]

Project: IMPlementation of evidence based facility and community interventions to reduce the treatment gap for depRESSion (IMPRESS)

Conflicts of interest

Language

English

Country
England

Stage of review
Review Ongoing

Subject index terms status
Subject indexing assigned by CRD

Subject index terms
Humans; Mental Disorders; Psychotherapy

Date of registration in PROSPERO
19 July 2021

Date of first submission
11 July 2021

Stage of review at time of this submission

**Stage**	**Started**	**Completed**
Preliminary searches	Yes	No
Piloting of the study selection process	No	No
Formal screening of search results against eligibility criteria	No	No
Data extraction	No	No
Risk of bias (quality) assessment	No	No
Data analysis	No	No

The record owner confirms that the information they have supplied for this submission is accurate and complete and they understand that deliberate provision of inaccurate information or omission of data may be construed as scientific misconduct.
The record owner confirms that they will update the status of the review when it is completed and will add publication details in due course.

Versions
19 July 2021
