# Supplementary material for: Interventions for improving adherence to psychological treatments for common mental disorders: a systematic review
Source: Glob Ment Health (Camb). 2024 Oct 17;11:e83. doi: 10.1017/gmh.2024.94 (PMC11504929; doi:10.1017/gmh.2024.94)
Supplement: Biswal et al. supplementary material 2 — Biswal et al. supplementary material [file S2054425124000943sup002.docx]

**Appendix 1: Search Strategy (Medline)**

1. Mood Disorders/

2. Depressive Disorder/

3. Depressive Disorder, Major/

4. Depressive Disorder, Treatment-resistant/

5. Dysthymic Disorder/

6. Neurotic Disorders/

7. Adjustment Disorders/

8. Anxiety Disorders/

9. Phobic Disorders/

10. Panic Disorder/

11. Somatoform Disorders/

12. Conversion Disorder/

13. Depression/

14. Anxiety/

15. Anxiety, Separation/

16. Agoraphobia/

17. Phobia, Social/

18. Panic/

19. Hypochondriasis/

20. Neurasthenia/

21. Neurocirculatory Asthenia/

22. Affective Symptoms/

23. ((mood or affect* or adjust* or conversion or somatic) adj2 disorder*).ti,ab,hw,kf,kw.

24. (depress* or MDD).ti,ab,hw,kf,kw.

25. (anx* or GAD).ti,ab,hw,kf,kw.

26. (common mental disorder* or CMD*).ti,ab,hw,kf,kw.

27. dysthym*.ti,ab,hw,kf,kw.

28. (neurotic* or neuros*).ti,ab,hw,kf,kw.

29. (phobi* or acrophob* or agoraphob* or claustrophob* or emetophob* or kinesiophob* or neophob* or trypanophob* or sociophob*).ti,ab,hw,kf,kw.

30. panic*.ti,ab,hw,kf,kw.

31. (somatoform or somati#ation).ti,ab,hw,kf,kw.

32. hypochondria*.ti,ab,hw,kf,kw.

33. (neurastheni* or neurocirculatory astheni*).ti,ab,hw,kf,kw.

34. 1 or 2 or 3 or 4 or 5 or 6 or 7 or 8 or 9 or 10 or 11 or 12 or 13 or 14 or 15 or 16 or 17 or 18 or 19 or 20 or 21 or 22 or 23 or 24 or 25 or 26 or 27 or 28 or 29 or 30 or 31 or 32 or 33

35. Patient Compliance/

36. "Treatment Adherence and Compliance"/

37. (adher* or adheren* or nonadheren*).ti,ab,hw,kf,kw.

38. (comply* or complie* or complian* or noncomplian*).ti,ab,hw,kf,kw.

39. (concordan* or nonconcordan*).ti,ab,hw,kf,kw.

40. ((treatment or therapy or program* or service* or patient or homework or appointment) adj4 (adher* or adheren* or nonadheren*)).ti,ab,hw,kf,kw.

41. ((treatment or therapy or program* or service* or patient or homework or appointment) adj4 (comply* or complie* or complian* or noncomplian*)).ti,ab,hw,kf,kw.

42. ((treatment or therapy or program* or service* or patient) adj4 (concordan* or nonconcordan*)).ti,ab,hw,kf,kw.

43. ((treatment or therapy or program* or service*) adj4 (persisten* or nonpersisten* or interrupt*)).ti,ab,hw,kf,kw.

44. ((treatment or therapy or program* or service* or appointment) adj4 (attend* or nonattendan*)).ti,ab,hw,kf,kw.

45. ((treatment or therapy or program* or service*) adj4 (continu* or discontinu*)).ti,ab,hw,kf,kw.

46. ((treatment or therapy or program* or service* or patient) adj4 participat*).ti,ab,hw,kf,kw.

47. ((treatment or therapy or program* or service* or patient) adj4 (engag* or disengag*)).ti,ab,hw,kf,kw.

48. ((treatment or therapy or program* or service*) adj4 retention).ti,ab,hw,kf,kw.

49. ((treatment or therapy or service*) adj4 utili#*).ti,ab,hw,kf,kw.

50. ((treatment or therapy or patient) adj4 motivat*).ti,ab,hw,kf,kw.

51. ((treatment or therapy or program* or homework) adj4 complet*).ti,ab,hw,kf,kw.

52. (treatment adj2 (integrity or fidelity)).ti,ab,hw,kf,kw.

53. (premature* adj2 (discontinu* or terminat*)).ti,ab,hw,kf,kw.

54. ((adheren* or nonadheren* or non-adheren*) adj behavio?r*).ti,ab,hw,kf,kw.

55. ((complian* or noncomplian* or non-complian*) adj behavio?r*).ti,ab,hw,kf,kw.

56. 35 or 36 or 37 or 38 or 39 or 40 or 41 or 42 or 43 or 44 or 45 or 46 or 47 or 48 or 49 or 50 or 51 or 52 or 53 or 54 or 55

57. exp Psychotherapy/

58. exp Counseling/

59. exp Sensory Art Therapies/

60. exp Mind-Body Therapies/

61. Transtheoretical Model/

62. Personal Construct Theory/

63. (psychotherap* or psychoanaly*).ti,ab,hw,kf,kw.

64. (behavio?r* adj (therap* or modification)).ti,ab,hw,kf,kw.

65. (cognitive behavio?r* therap* or CBT).ti,ab,hw,kf,kw.

66. (dialectical behavio?r* therap* or DBT).ti,ab,hw,kf,kw.

67. ((cognitive or psychodynamic or humanistic or integrative or systemic or mind-body or expressive or art?) adj therap*).ti,ab,hw,kf,kw.

68. (psychological adj (treatment or intervention or therap*)).ti,ab,hw,kf,kw.

69. (psychosocial adj (treatment or intervention or therap*)).ti,ab,hw,kf,kw.

70. (psychiatric adj (treatment or intervention)).ti,ab,hw,kf,kw.

71. (mental health adj (treatment or intervention)).ti,ab,hw,kf,kw.

72. activity schedul*.ti,ab,hw,kf,kw.

73. (aversi* adj (therapy or conditioning)).ti,ab,hw,kf,kw.

74. covert sensiti#ation.ti,ab,hw,kf,kw.

75. behavio?r* contracting.ti,ab,hw,kf,kw.

76. (biofeedback or sensory feedback).ti,ab,hw,kf,kw.

77. ((contingency or stress) adj management).ti,ab,hw,kf,kw.

78. ((distraction or exposure or abreaction or implosive or problem focused or social effectiveness or group or "acceptance and commitment" or reality or self management or self control or problem solving or metacognitive or insight oriented or psychoanalytic or individual or alderian or schema or schema focused or person centered or person centred or client centered or client centred or Rogerian or existential or experiential or Gestalt or nondirective or supportive or cognitive analytic or eclectic or multimodal or transtheoretical or couples or marital or relationship or family or emotion focused or emotionally focused or narrative or socioenvironmental or milieu or age regression or dance or dance movement or drama or music or play or primal or sex or reminiscence or Morita or persuasion) adj therapy).ti,ab,hw,kf,kw.

79. systematic desensiti#ation.ti,ab,hw,kf,kw.

80. (eye movement desensiti#ation reprocessing or EMDR).ti,ab,hw,kf,kw.

81. (pleasant adj (event* or activit*)).ti,ab,hw,kf,kw.

82. psychoeducation*.ti,ab,hw,kf,kw.

83. reciprocal inhibition.ti,ab,hw,kf,kw.

84. ((relaxation or emotional freedom) adj technique*).ti,ab,hw,kf,kw.

85. ((autogenic or assertiveness or sensitivity or compassionate mind) adj training).ti,ab,hw,kf,kw.

86. guided imagery.ti,ab,hw,kf,kw.

87. response cost.ti,ab,hw,kf,kw.

88. chronotherapy.ti,ab,hw,kf,kw.

89. (social skills training or SST).ti,ab,hw,kf,kw.

90. (rational emotive therapy or rational emotive behavio?r* therapy or REBT).ti,ab,hw,kf,kw.

91. (cognitive adj (restructuring or reframing)).ti,ab,hw,kf,kw.

92. role play*.ti,ab,hw,kf,kw.

93. behavio?r* activation.ti,ab,hw,kf,kw.

94. (cognitive behavio?ral analysis system of psychotherapy or CBASP).ti,ab,hw,kf,kw.

95. (compassion focused therapy or CFT).ti,ab,hw,kf,kw.

96. functional analytic.ti,ab,hw,kf,kw.

97. (mindfulness or MBCT or MBSR).ti,ab,hw,kf,kw.

98. (transference or countertransference).ti,ab,hw,kf,kw.

99. (defusion or deliterali#ation).ti,ab,hw,kf,kw.

100. (jungian or kleinian).ti,ab,hw,kf,kw.

101. (object relations adj (theory or therapy)).ti,ab,hw,kf,kw.

102. (dream adj (analysis or therapy)).ti,ab,hw,kf,kw.

103. self analysis.ti,ab,hw,kf,kw.

104. free association.ti,ab,hw,kf,kw.

105. process experiential.ti,ab,hw,kf,kw.

106. (griefwork or grief work).ti,ab,hw,kf,kw.

107. transactional analysis.ti,ab,hw,kf,kw.

108. counsel?ing.ti,ab,hw,kf,kw.

109. (integrative behavio?ral couple? therapy or IBCT).ti,ab,hw,kf,kw.

110. (interpersonal therapy or IPT).ti,ab,hw,kf,kw.

111. (transtheoretical model or TTM).ti,ab,hw,kf,kw.

112. (co-therapy or conjoint therapy).ti,ab,hw,kf,kw.

113. (personal construct adj (theory or therapy)).ti,ab,hw,kf,kw.

114. therapeutic communit*.ti,ab,hw,kf,kw.

115. (solution focused therapy or solution focused brief therapy or SFBT).ti,ab,hw,kf,kw.

116. bibliotherapy.ti,ab,hw,kf,kw.

117. catharsis.ti,ab,hw,kf,kw.

118. crisis intervention.ti,ab,hw,kf,kw.

119. meditat*.ti,ab,hw,kf,kw.

120. psychodrama.ti,ab,hw,kf,kw.

121. (hypnotherapy or hypnosis).ti,ab,hw,kf,kw.

122. 57 or 58 or 59 or 60 or 61 or 62 or 63 or 64 or 65 or 66 or 67 or 68 or 69 or 70 or 71 or 72 or 73 or 74 or 75 or 76 or 77 or 78 or 79 or 80 or 81 or 82 or 83 or 84 or 85 or 86 or 87 or 88 or 89 or 90 or 91 or 92 or 93 or 94 or 95 or 96 or 97 or 98 or 99 or 100 or 101 or 102 or 103 or 104 or 105 or 106 or 107 or 108 or 109 or 110 or 111 or 112 or 113 or 114 or 115 or 116 or 117 or 118 or 119 or 120 or 121

123. 4

124. Randomized Controlled Trial/

125. exp Randomized Controlled Trials as Topic/

126. Controlled Clinical Trial/

127. exp Controlled Clinical Trials as Topic/

128. Randomization/

129. Random Allocation/

130. Double-Blind Method/

131. Double-Blind Studies/

132. Single-Blind Method/

133. Single-Blind Studies/

134. Placebos/

135. Control Groups/

136. Control Group/

137. (random* or sham or placebo*).ti,ab,hw,kf,kw.

138. ((singl* or doubl*) adj (blind* or dumm* or mask*)).ti,ab,hw,kf,kw.

139. ((tripl* or trebl*) adj (blind* or dumm* or mask*)).ti,ab,hw,kf,kw.

140. (control* adj3 (study or studies or trial* or group*)).ti,ab,hw,kf,kw.

141. (nonrandom* or non-random* or quasi-random*).ti,ab,hw,kf,kw.

142. allocated.ti,ab,hw.

143. ((open label or open-label) adj5 (study or studies or trial*)).ti,ab,hw,kf,kw.

144. ((equivalence or superiority or non-inferiority or noninferiority) adj3 (study or studies or trial*)).ti,ab,hw,kf,kw.

145. (pragmatic study or pragmatic studies).ti,ab,hw,kf,kw.

146. ((pragmatic or practical) adj3 trial*).ti,ab,hw,kf,kw.

147. (phase adj3 (III or "3") adj3 (study or studies or trial*)).ti,hw,kf,kw.

148. 123 or 124 or 125 or 126 or 127 or 128 or 129 or 130 or 131 or 132 or 133 or 134 or 135 or 136 or 137 or 138 or 139 or 140 or 141 or 142 or 143 or 144 or 145 or 146 or 147

149. 34 and 56 and 122 and 148
