# Supplementary material for: Interventions for improving adherence to psychological treatments for common mental disorders: a systematic review
Source: Glob Ment Health (Camb). 2024 Oct 17;11:e83. doi: 10.1017/gmh.2024.94 (PMC11504929; doi:10.1017/gmh.2024.94)
Supplement: Biswal et al. supplementary material 3 — Biswal et al. supplementary material [file S2054425124000943sup003.docx]

**Appendix 2: Table showing components of evaluated interventions**

| **Author Name, Year** | Session reminders | Content reminder | Supportive Monitoring | Feedback | Motivational Interviewing | Therapy orientation | Case  management | Telephone based therapy | Placebo | Implementation Intention |
| --- | --- | --- | --- | --- | --- | --- | --- | --- | --- | --- |
| *Aguilera et al., 2017* |  | **✓** |  |  |  |  |  |  |  |  |
| *Alfonsson et al., 2019* | **✓** | **✓** |  |  |  |  |  |  |  |  |
| *Avishai et al., 2018* |  |  |  |  |  |  |  |  |  | **✓** |
| *Barrera et al., 2016* |  |  |  |  | **✓** |  |  |  |  |  |
| *Clough et al., 2014* | **✓** |  |  |  |  |  |  |  |  |  |
| *Daley et al., 1998* |  |  |  |  | **✓** |  |  |  |  |  |
| *Delgadillo et al., 2015* | **✓** |  |  |  |  |  |  |  |  |  |
| *Delgadillo et al., 2017* |  | **✓** |  |  |  | **✓** |  |  |  |  |
| *Furber et al., 2014* | **✓** | **✓** |  |  |  |  |  |  |  |  |
| *Hoehn-Saric et al., 1964* |  |  |  |  |  | **✓** |  |  |  |  |
| *Jurinec & Schienle, 2020* |  |  |  |  |  |  |  |  | **✓** |  |
| *Latour & Cappeliez, 1994* |  |  |  |  |  | **✓** |  |  |  |  |
| *Miranda et al., 2003* |  |  | **✓** |  |  |  | **✓** |  |  |  |
| *Mohr et al., 2012* |  |  |  |  |  |  |  | **✓** |  |  |
| *Perez et al., 2021* |  | **✓** | **✓** | **✓** |  |  |  |  |  |  |
| *Peters et al., 2019* |  |  |  |  | **✓** |  |  |  |  |  |
| *Raue et al., 2019* |  |  | **✓** |  |  |  | **✓** |  |  |  |
| *Reis & Brown, 2006* |  |  |  |  |  | **✓** |  |  |  |  |
| *Stein et al., 2020* |  |  |  |  |  | **✓** |  |  |  |  |
| *Wang et al., 2022* |  |  |  |  |  | **✓** |  |  |  |  |
| *Wells et al., 2019* | **✓** | **✓** |  |  |  |  |  |  |  |  |
| *Westra & Dozois, 2006* |  |  |  |  | **✓** |  |  |  |  |  |
| *Westra et al., 2009* |  |  |  |  | **✓** |  |  |  |  |  |
