# Supplementary material for: Interventions for improving adherence to psychological treatments for common mental disorders: a systematic review
Source: Glob Ment Health (Camb). 2024 Oct 17;11:e83. doi: 10.1017/gmh.2024.94 (PMC11504929; doi:10.1017/gmh.2024.94)
Supplement: Biswal et al. supplementary material 4 — Biswal et al. supplementary material [file S2054425124000943sup004.docx]

**Appendix 3: Quality of Reporting of Included Studies in the Review**

1. Quality assessments conducted using RoB2 tool for RCTs.

| **Author, Year of Publication** | **Domain 1:**  Randomization | **Domain 2:**  Deviations from Intended Interventions | **Domain 3:**  Missing Outcome Data | **Domain 4:**  Measurement of the outcome | **Domain 5:**  Selection of the reported result | **Domain 6:**  Overall Bias |
| --- | --- | --- | --- | --- | --- | --- |
| Avishai et al., 2018 | Low | Low | Low | Low | Some concerns | Low |
| Alfonsson, 2019 | Low | Low | Low | Low | Low | Low |
| Barrera et al., 2016 | Low | Low | Low | Low | Some concerns | Low |
| Clough, 2014 | Low | Low | Low | Low | Some concerns | Low |
| Delgadillo et al., 2015 | Low | Low | Low | Low | Low | Low |
| Hoehn-Saric et al., 1964 | Low | Low | Low | Low | Low | Low |
| Jurinec et al., 2020 | Low | Low | Low | Low | Low | Low |
| Mohr et al., 2012 | Low | Low | Low | Low | Low | Low |
| Peters et al., 2019 | Low | Some concerns | Low | Low | Low | Some concerns |
| Raue et al., 2019 | Low | Low | Low | Some concerns | Low | Some concerns |
| Stein et al., 2020 | Low | Low | High | Low | Some concerns | High |
| Westra et al., 2006 | Low | Low | Low | Low | Low | Low |
| Westra et al., 2009 | Low | High | Low | Low | Low | High |
| Latour et al., 1994 | Low | Low | Low | Low | Low | Low |
| Reis et al., 2006 | Some concerns | Low | Low | High | Low | High |
| Miranda et al., 2003 | Low | Low | Low | Low | Low | Low |
| Perez et al., 2019 | Low | Low | Low | Low | Some concerns | Low |
| Wang et al., 2022 | High | Low | Low | Low | Some concerns | High |

1. Quality assessments conducted using ROBINS-I for nRCTs

| **Author, Year of Publication** | **Domain 1:**  Confounding | **Domain 2:**  Selection of participants into the study | **Domain 3:**  Classification of interventions | **Domain 4:**  Deviations from intended interventions | **Domain 5:**  Missing data | **Domain 6:**  Measurement of the outcome | **Domain 7:**  Selection of the reported result |
| --- | --- | --- | --- | --- | --- | --- | --- |
| Aguilera et al., 2017 | Moderate | Low | Low | Low | Low | Low | Low |
| Daley et al., 1998 | NI | Low | Low | NI | Low | NI | Low |
| Delgadillo et al., 2017 | Low | Low | Low | Low | Low | Low | Low |
| Furber et al., 2014 | Moderate | Low | Low | Low | Low | Low | Low |
| Wells et al., 2019 | Moderate | Low | Low | Low | Low | Low | Low |

**Note:**
High indicates High Risk
Some Concerns indicates Moderate Risk
Low indicates Low Risk
NI indicates “Not enough information”
