# Supplementary material for: Interventions for improving adherence to psychological treatments for common mental disorders: a systematic review
Source: Glob Ment Health (Camb). 2024 Oct 17;11:e83. doi: 10.1017/gmh.2024.94 (PMC11504929; doi:10.1017/gmh.2024.94)
Supplement: Peer review history

# Editor report: Recommendation: Interventions for improving adherence to psychological treatments for common mental disorders: a systematic review — R0/PR2

**DOI:** 10.1017/gmh.2024.94.pr2

**Affiliations:** University of KwaZulu-Natal, South Africa

Please would you attend to reviewer suggestions made by reviewer 2.

# Editor report: Decision: Interventions for improving adherence to psychological treatments for common mental disorders: a systematic review — R0/PR3

**DOI:** 10.1017/gmh.2024.94.pr3

**Affiliations:** Johns Hopkins University Bloomberg School of Public Health, United States

No accompanying comment.

# Editor report: Recommendation: Interventions for improving adherence to psychological treatments for common mental disorders: a systematic review — R1/PR5

**DOI:** 10.1017/gmh.2024.94.pr5

**Affiliations:** University of KwaZulu-Natal, South Africa

No accompanying comment.

# Editor report: Decision: Interventions for improving adherence to psychological treatments for common mental disorders: a systematic review — R1/PR6

**DOI:** 10.1017/gmh.2024.94.pr6

**Affiliations:** Johns Hopkins University Bloomberg School of Public Health, United States

No accompanying comment.
